# Light chain myeloma and detection of free light chains in serum and urine of dogs and cats

**DOI:** 10.1111/jvim.16070

**Published:** 2021-02-26

**Authors:** Robert Adam Harris, Matthew Miller, Dillon Donaghy, Laura Ashton, Catherine Langston, Todd Shockey, A Russell Moore

**Affiliations:** ^1^ Department of Microbiology, Immunology, and Pathology College of Veterinary Medicine and Biomedical Science, Colorado State University Fort Collins Colorado USA; ^2^ Department of Veterinary Clinical Sciences The Ohio State University Columbus Ohio USA; ^3^ Parkersburg Veterinary Hospital Parkersburg West Virginia USA

**Keywords:** Bence‐Jones proteins, electrophoresis, gammopathy, immunoglobulin, proteinuria

## Abstract

**Background:**

Detection of free light chains (fLC) in animals relies on protein electrophoresis or the Bence‐Jones protein test on urine.

**Objective:**

To describe the detection of both serum fLC (sfLC) and urine fLC (ufLC) in 8 dogs and 2 cats using a commercially available human immunofixation (IF) kit.

**Animals:**

Archived serum or urine samples from 27 dogs and 2 cats submitted to the Colorado State University Veterinary Diagnostic Laboratory for routine diagnostics.

**Methods:**

Retrospective study evaluating the presence of fLC in dogs and cats using agarose gel electrophoresis and routine and fLC IF performed on serum and urine. The performance of the fLC IF reagents was evaluated using samples characterized by routine IF, tandem mass spectrometry, and a combination of fLC IF and western blotting. Free light chains were documented by paired electrophoresis and fLC IF.

**Results:**

The fLC only myeloma case developed end‐stage renal failure 5 months post initial diagnosis. All electrophoresis‐defined urinary Bence‐Jones proteins were labeled by the anti‐free λ light chain (anti‐fλ) reagent; none were labeled by the anti‐free κ light chain (anti‐fκ); 2 of these were identified as fκ by mass spectrometry. An electrophoretically identical protein restriction that was labeled by the anti‐fλ reagent was present in the paired serum from 5/8 of cases, documenting sfLC.

**Conclusions and Clinical Importance:**

Commercially available human IF reagents identified sfLC and ufLC in both dogs and cats. Free light chains may be nephrotoxic in dogs.

**Abbreviations**
AGE: agarose gel electrophoresis
fLC: free light chains
fκ/fλ: free kappa/lambda light chains
GAM: IgG, IgA, and IgM
IF: immunofixation
LC‐MS/MS: liquid chromatography and tandem mass spectrometry
RI: reference interval
sfLC: serum free light chains
SPE: serum protein electrophoresis
ufLC: urine free light chains
UP : UC: urine protein : urine creatinine
UPE: urine protein electrophoresis
κ/λ‐LC: kappa/lambda‐light chain
nrPAGE: nonreduced lithium‐dodecyl‐sulfate polyacrylamide gel electrophoresis
WS: whole serum

## 1 INTRODUCTION

Immunoglobulin light chains (LC) are typically overproduced compared with immunoglobulin heavy chains by both normal and neoplastic B‐cell lymphocytes and plasma cells. These unbound or free light chains (fLC) are approximately 20 to 30 kDa and are freely filtered from the serum into the urine.^1^ For this reason, fLC are typically found in low concentration in serum. Accumulation of these proteins in serum is dependent on the amount of fLC being produced and glomerular filtration capacity.^1^, ^2^ Monoclonal fLCs present in urine are referred to as Bence‐Jones proteins.

LC myeloma is a variation of multiple myeloma where the clonal plasma cells secrete only fLCs.^2^, ^3^ Few case reports of LC myeloma are published in veterinary medicine.^4^, ^5^ In human medicine, fLC detection is imperative for diagnosis and monitoring of the response to treatment.^6^ fLCs also aid in prognosis for human cases as they are nephrotoxic.^7^

We describe the clinical progression of a dog with LC myeloma that was identified using urine and serum agarose gel electrophoresis (AGE) and commercially available anti‐human fLC immunofixation (IF) reagents. The performance of the anti‐human fLC IF reagents is further characterized.

## 2 CASE 1 HISTORY

An 8‐year‐old female spayed outdoor‐only mixed breed dog, without travel history or known exposure to toxic agents, was diagnosed with prerenal azotemia on preanesthetic blood work based on an elevated serum creatinine concentration (2.0 mg/dL, reference interval [RI]: 0.4‐1.3 mg/dL) and urine specific gravity (USG) of 1.032. All other values on routine CBC and chemistry were within normal limits. Amoxicillin‐clavulanic acid (unknown dose) was administered for 1 week because of suspected urinary tract infection but was discontinued after aerobic and anaerobic urine cultures had no growth. The dog's azotemia worsened (creatinine 2.6 mg/dL; blood urea nitrogen (BUN) 33 mg/dL, RI: 10‐27 mg/dL; and symmetric dimethylarginine (SDMA) increased from 13 μg/dL prior to treatment to 17 μg/dL, RI: 0‐14 μg/dL). These values remained static (creatinine 2.0 mg/dL, BUN 9 mg/dL, SDMA 17 μg/dL) after 3 days of fluid treatment administered IV. The dog was transitioned to a renal diet (Hill's *k*/*d*, Hill's Pet Nutrition, Inc., Topeka, Kansas) and remained clinically normal, without evidence of vomiting or diarrhea or changes in water intake or urination. However, 1 month after initial detection of azotemia there was progression (creatinine 3.7 mg/dL; BUN 37 mg/dL; SDMA 26 μg/dL; phosphorous 4.7 mg/dL, RI: 3.2‐8.1 mg/dL; potassium 6.6 mEq/L, RI: 4.2‐5.4 mEq/L; and USG 1.019). The dog was referred to the Ohio State Veterinary Medical Center.

On intake physical examination, the dog was quiet, alert, and responsive and had evidence of mild discomfort on abdominal palpation, but no other abnormalities were detected. Venous blood pH was 7.4 (RI: 7.38‐7.48), creatinine 4.1 mg/dL (RI: 0.4‐1.3 mg/dL), BUN 35 mg/dL (RI: 10‐27 mg/dL), and potassium 5.08 mEq/L (RI: 3.9‐5.5 mEq/L). All other values were within RI. In addition, USG was 1.020, protein 1+, pH 5.0, and there was an inactive urine sediment examination. The PCV was 42% (RI: 35‐55%) and total solids (TS) 8.0 g/dL (RI: 5.6‐7.3 g/dL). The blood pressure and urine protein : creatinine (UP : UC) were 150 mm Hg and 0.95, respectively.

Abdominal ultrasound revealed a 6 × 5 × 4.5 cm heteroechoic mass arising from the caudal margin of the left liver as well as a 5.8 mm hypoechoic nodule within the left liver. A 1.1 cm heterogeneously hypoechoic nodule and a 4 mm hypoechoic nodule were present in the spleen. A single left renal cortical cyst measuring 4 mm was present. An aspiration biopsy specimen of the liver mass was obtained. Hepatocytes were not identified but there was a population of round cells characterized by a moderate to high nucleus to cytoplasm ratio, with mild to moderate anisocytosis and anisokaryosis. Their cytologic characteristics, including their degree of cytoplasmic basophilia, eccentric nucleus, binucleation, and paranuclear clear zone, were compatible with a plasma cell neoplasm.

**FIGURE 1:**
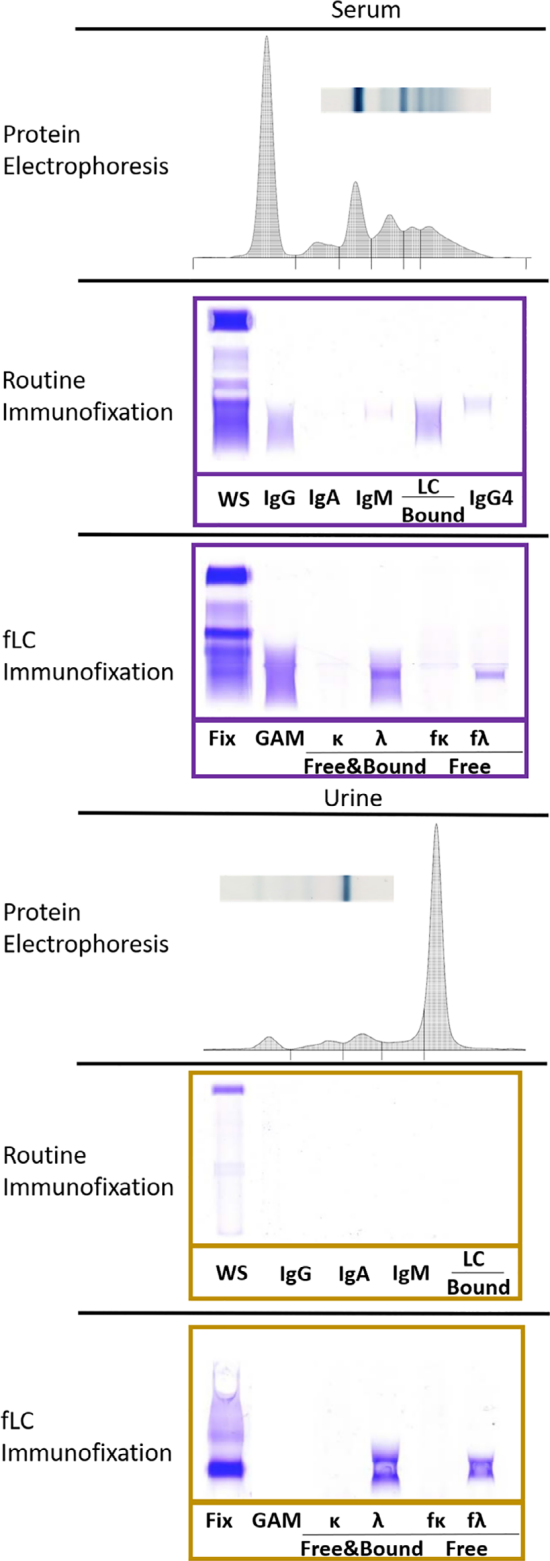
SPE, routine IF and fLC IF from case 1. Serum protein electrophoretic tracing and gel were within normal limits on initial review. Urine protein electrophoresis revealed a restricted band and a monoclonal spike. fLC, free light chains; IF, immunofixation; SPE, serum protein electrophoresis

The next day, the dog was evaluated for evidence of metastasis. Aspiration biopsy specimens of the normoechoic liver parenchyma and spleen were obtained. Bone marrow aspiration for cytologic evaluation was also performed. The liver parenchyma was cytologically normal. The spleen showed evidence of scant extramedullary hematopoiesis and suspected aspiration of a lymphoid follicle, based on the primary nucleated cell types being a heterogenous lymphoid population. Plasma cells within the splenic aspirate were considered normal. The bone marrow aspirate showed cytologically normal proportions and morphology of the erythroid, myeloid, megakaryocytic, and lymphoid populations. Plasma cells accounted for approximately 4% of the nucleated cells within the aspirate, which was interpreted as mild plasma cell hyperplasia. There was a small amount of abdominal effusion. Examination of an aspirate of this effusion was consistent with a hemorrhagic effusion (total protein 4.8 g/dL, red blood cells (RBC) 4.00 × 10^6^/μL). Prothrombin time and partial thromboplastin time (PT/PTT) was considered normal. Abnormalities were not detected on thoracic radiographs; however, the skeletal system was not radiologically examined. Repeat serum biochemistry showed mild improvement of the dog's azotemia (creatinine 3.7 mg/dL; BUN 33 mg/dL; phosphorous 4.0 mg/dL, RI: 2.2‐6.3 mg/dL; potassium 5.4 mEq/L; albumin 3.2 g/dL, RI: 3.3‐4.2 g/dL; cholesterol 363 mg/dL fasted, RI: 122‐345 mg/dL; and creatine kinase 450 IU/L, RI: 53‐372 IU/L). All other values were within normal limits. A urine protein electrophoresis (UPE) and a serum protein electrophoresis (SPE) were performed. Urine and serum electrophoretic and IF techniques are discussed in Section 3. These tests indicated the presence of fLC in the urine and serum, and a monoclonal heavy chain was not documented in either location (Figure 1 and Table S1).

The dog's PCV/TS initially declined during hospitalization, likely due to the hemorrhagic effusion and fluid treatment. At discharge, the PCV/TS was stable at 34% and 6.6 g/dL. Blood pressure at discharge was consistently 180 mmHg; amlodipine was administered at 3.75 mg (0.12 mg/kg) daily. The dog was discharged and prescribed maropitant 2 mg/kg daily (Cerenia, Zoetis, Parsippany, New Jersy) and capromorelin 3.1 mg/kg daily (Entyce, Aratana Therapeutics Inc, Leawood, Kansas). One week after discharge, a liver lobectomy to remove the solitary liver mass and collect hepatic lymph node and renal biopsy specimens were performed. No complications were noted during or after surgery. Histopathology confirmed the liver mass was an extramedullary plasma cell tumor. The kidney had multifocal areas of tubular loss and dilated cystic tubules, consistent with end‐stage renal disease, accompanied by chronic tubulointerstitial lymphoplasmacytic nephritis. These lesions were evidence of end‐stage renal disease. Hepatic lymph nodes did not have evidence of metastatic disease, as normal cortical and medullary architecture were maintained and no overtly neoplastic plasma cells were identified. Chemotherapy was not pursued. The dog's BUN (min‐max 49‐130, RI: 7‐27 mg/dL), creatinine (min‐max 3.2‐13.6, RI: 0.5‐1.8 mg/dL), and SDMA (min‐max 13‐59, RI: 0‐14 μg/dL) were consistently abnormal. Approximately 5 months after surgical excision, the dog, was euthanized due to worsening clinical signs, including anorexia, vomiting, and lethargy, associated with end‐stage renal failure. At the time of euthanasia, the dog's renal function had markedly declined (BUN >130 mg/dL, Creatinine >13.6 mg/dL, SDMA 59 μg/dL).

Based on all available data, the dog was diagnosed with a LC multiple myeloma. The performance of the anti‐human fLC kit to identify canine fLC was further evaluated using archived sera and urine.

## 3 MATERIALS AND METHODS

### 3.1 Animals

The use of animal samples complied with institutional policies and owner consent. Samples were not solicited for this study. Laboratory identification numbers were used to ensure proper identification.

The index case and 28 archived samples were evaluated. This included 10 cases (8 dogs and 2 cats) identified as having serum fLC (sfLC), urine fLC (ufLC), or both, of which 8 cases had paired urine and serum and 2 cases (both dogs) had serum only. The remaining dogs had a monoclonal or biclonal gammopathy and sufficient archived serum to perform liquid chromatography and tandem mass spectrometry (LC‐MS/MS) to characterize their LCs. The heavy chains in these dogs had been characterized previously.^8^ Serum from 2 healthy dogs without B‐cell lymphoid neoplasia were also used. All samples were submitted to the clinical pathology laboratory at Colorado State University and stored at −80°C until evaluation. Clinical history and additional diagnostics were recorded from the Clinical Pathology lab submission form and available records.

### 3.2 AGE protein electrophoresis

Serum and urine samples were assessed under neat conditions, except in cases where the urine protein was below 1 g/dL. In these cases, the urine was concentrated to >1 g/dL (Vivapore 5, Sartorius Stredim Lab Ltd, Gloucestershire, UK). Serum samples were evaluated using a biuret total protein (Cobas c501 Roche Diagnostics, Indianapolis, Indiana) and urine samples were evaluated using a benzethonium chloride assay and creatinine (Cobas c501). Protein electrophoresis using AGE was performed and gels were stained with amido black (Sebia Hydrasys with Hydragel Protein [E] with amido black kit, Sebia, France), as previously described.^9^ Urinary fLC concentration was determined using the method previously validated for serum M‐protein. Electrophoretic tracings and gels were reviewed by 2 reviewers (R.A.D.H. and AR.M.).

### 3.3 Immunofixation

Immunofixation was performed on all samples using the Hydragel IF/Bence Jones kit (Sebia, France) and commercially available labeling antibodies. Canine IF targeted whole serum (WS), IgG‐FC, IgG4, IgA and IgM heavy chains and LC in a validated protocol (see Table S2).^8^, ^10^, ^11^ Feline IF used an identical protocol and feline‐specific reagents that targeted WS, IgG, IgA, and IgM heavy chains and LC (see Table S2).

Immunofixation for fLC was performed using a fLC reagent set (Sebia Antisera K & L free light chains [PN 4836]) on all canine and feline samples, according to the manufacturer's instructions.^12^ These reagents include a nonspecific protein fixative (fix) and antibody based reagents which targets human IgG/IgA/IgM (GAM) heavy chain, human free and bound κ‐LC (anti‐κ), human free and bound λ‐LC (anti‐λ), human free kappa LC (anti‐fκ), and human free lambda LC (anti‐fλ). In addition, the anti‐κ and anti‐fκ reagents had addition of 20 g/L polyethylene glycol 6000 to aid in antibody labeling, as previously described.^8^

### 3.4 Polyacrylamide gel electrophoresis and LC‐MS/MS

Serum or urine proteins from 3 patients were evaluated by polyacrylamide gel electrophoresis (PAGE) under nonreducing conditions for confirmation of fLC presence (nrPAGE, fLC containing samples) or reducing conditions (rPAGE, remaining samples), as previously described.^8^ Briefly, a standard 10 ng of protein from all samples were electrophoresed on NuPAGE Bis‐Tris Precast Gels (Invitrogen, Life Technologies Corporation, Carlsbad, California) and visualized using Coomassie blue stain. The prominent 25 to 30 kDa protein was excised and identified using LC‐MS/MS as previously described.^8^ Briefly, mass spectra results and peptide sequences charge states were deconvoluted and deisotoped by ProteoWizard (ProteoWizard MsConvert, version 3.0). Spectra from all samples were searched using Mascot (Matrix Science, London, UK; version 2.6.0) against a common contaminants database (common Repository of Adventitious Proteins [cRAP]) and a reverse concatenated database containing *Canis familiaris* or *Felis catus* reference sequences (National Center for Biotechnology Information, [NCBI]) plus immunoglobulin entries from NCBI and international ImMunoGeneTics information system (IMGT), assuming trypsin digestion. Search results from samples were imported and combined using the probabilistic protein identification algorithms implemented in the Scaffold software (version Scaffold_4.8.4, Proteome Software Inc., Portland, Oregon).^13^, ^14^ Peptide thresholds were set (90%) such that a peptide false discovery rate (FDR) of 0.00% was achieved based on hits to the reverse database.^15^ Protein identifications were accepted if they could be established at greater than 95.0% probability and contained at least 2 identified peptides. Protein probabilities were assigned by the Protein Prophet algorithm.^16^ Proteins that contained similar peptides and could not be differentiated based on MS/MS analysis alone were grouped to satisfy the principles of parsimony. The identity of the submitted protein was defined as the immunoglobulin LC with the highest spectral count.

### 3.5 Western blot

Proteins within the serum and urine samples were quantified using the bicinchoninic acid assay. Equal amounts of protein were solubilized in Tris buffer containing 2% sodium dodecyl sulfate (SDS) and 10% glycerol. Samples were reduced in dithiothreitol and heated at 85°C for 8 to 10 minutes before loading. Proteins were separated using SDS‐PAGE, and transferred to a polyvinylidene fluoride membrane. The primary detection antibodies used for western blotting were provided in the Sebia Antisera K&L fLC. The secondary detection antibody was a horseradish peroxidase conjugated donkey anti‐rabbit antibody (ab6802) (Abcam, Cambridge, Massachusetts). Immunolabeling was visualized on a chemiluminescent imager (Bio‐Rad GelDoc, Lifescience, Hercules, California).

### 3.6 Limit of detection assay

Sample was available to evaluate the limit of detection of the anti‐fλ reagent but not anti‐fκ reagent. The concentration of fLC was quantified densitometrically in the urine of case #3 as this sample had the highest fLC percentage and absolute concentration.^9^ This urine sample was then spiked into serum from 2 healthy dogs to obtain a final fLC concentration of 0.29, 0.12, 0.05, 0.01, and 0.005 g/dL. Free LC IF using the anti‐fλ reagent was performed under routine conditions on each spiked sample and the nonspiked sample in a single run. The limit of detection was defined as the lowest dilution where anti‐fλ labeling could be visualized after gel imaging.

## 4 RESULTS

In the index case, case 1, both the serum total protein and globulin concentrations were within RIs. Serum globulin concentration remained within RI after surgery. Trace amounts of protein in the urine and mildly elevated UP : UC were present. Neither SPE nor routine IF revealed an M‐protein on initial review (Figure 1). The routine IF had appropriate polyclonal labeling of heavy chains and their bound LC. The urine protein electrophoretic tracing revealed a monoclonal spike within the γ‐globulin fraction. Routine IF of the urine sample was unable to identify an M‐protein. However, the anti‐fλ reagent identified a restricted protein band as fLC in both the urine and serum; documenting Bence‐Jones proteinuria and serum fLC.

**FIGURE 2:**
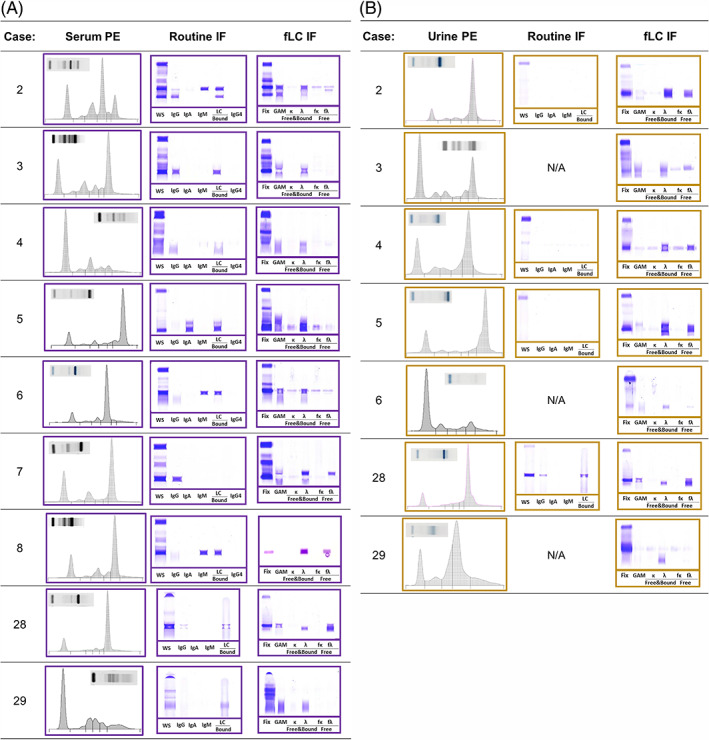
SPE, routine IF and fLC IF of 9 cases with serum and/or urine fLC. Seven patients had concurrently submitted urine samples. A, Serum samples are outlined with purple boxes. B, Urine samples are outlined with yellow boxes. fLC, free light chains; IF, immunofixation; SPE, serum protein electrophoresis

Electrophoretic tracings and gels, and routine and fLC IF gels from the 9 additional fLC cases are provided (Figure 2). A summary of the findings from all cases are listed, Table S1. Most of the fLC cases (6/9) had a monoclonal or biclonal gammopathy. One dog and 1 cat had only hypogammaglobulinemia documented by SPE and routine IF. Hypogammaglobulinemia was defined as an absolute γ‐globulin fraction equal to or less than the lower RI (0.33 g/dL) and a flattened electrophoretic morphology. Hypogammaglobulinemia in patients with an M‐protein in the γ‐globulin fraction were identified if they contained distinct restrictions that accounted for <50% of the fraction and the region surrounding the restriction was flattened and accounted for less than the lower limit of the γ‐globulin RI.

A prominent restricted protein band was noted in 5/7 samples by urine protein electrophoresis that was not present in the concurrent serum, suggesting Bence‐Jones proteinuria. UP : UC assessment was available for 6 of the cases with urinary fLC and considered elevated in all cases (average 2.50, min‐max 0.88‐3.68). Interestingly, case 1 had the lowest UP : UC. Urinary fLC concentration was calculated as absolute protein content (average 440.2 mg/dL, min‐max 6.4‐1390.9 mg/dL) and relative to creatinine excretion (M‐protein : UC average 1.08, min‐max 0.17‐1.65). The non‐fLC containing samples included 6 IgA biclonal gammopathies, 4 IgM monoclonal gammopathies and 9 IgG monoclonal gammopathies as documented by SPE and IF.

**FIGURE 3:**
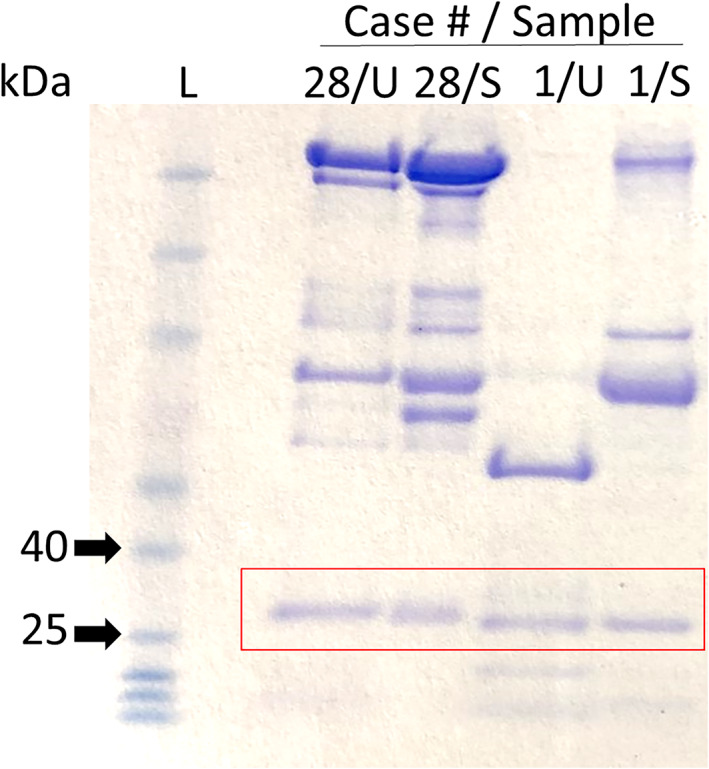
Nonreduced lithium‐dodecyl‐sulfate polyacrylamide gel electrophoresis (nrPAGE) to confirm the presence of free light chains (fLC): This is a representative nrPAGE (Coomassie Stained) experiment using feline (case 28) urine and serum and canine (case 1/index case) urine and serum samples. Restricted bands were identified between the 25‐ and 40‐kDa molecular markers. The red box indicates the targeted bands that were removed and submitted for tandem mass spectrometry

Paired nrPAGE evaluation of 2 dogs (cases 1 and 2) and 1 cat (case 8), documented a 25 to 30 kDa restricted band in both the serum and urine samples (Figure 3). Evaluation of the excised 25 to 30 kDa bands from 23 canine samples by LC‐MS/MS identified 10 κ and 13 λ LC, including 2 fλ and 2 fκ (Table S1). The single cat evaluated by LC‐MS/MS was identified as an fλ. Combining the IF determined heavy chain and LC‐MS/MS LC data, there were 5 IgA : κ, 1 IgA : λ, 1 IgM : κ, 3 IgM : λ, 2 IgG : κ, 7 IgG : λ, and 1 biclonal IgG : λ/M : λ dogs, and 1 IgG : λ cat.

To confirm that the anti‐human labeling antibodies were binding the excised protein from the PAGE gel, western blot assays using the anti‐λ, anti‐κ, anti‐fλ, anti‐fκ reagents were performed. Restricted bands were noted in the appropriate range, between the 20 and 30 kDa, using the ant‐fλ and anti‐fκ antibodies (Figure S1). Dense restricted bands were noted in cases 1, 2, and 9, confirmed to predominately have fλ by mass spectrometry, using the anti‐fλ antibody. A faint band was noted in case 12, predominately κ‐LC by mass spectrometry, using the anti‐fλ antibody. A dense band was identified in case 12 using the anti‐fκ antibody, and faint labeling was noted in all other cases. Similar labeling was noted by Western blot using the anti‐λ and anti‐κ reagents.

**FIGURE 4:**
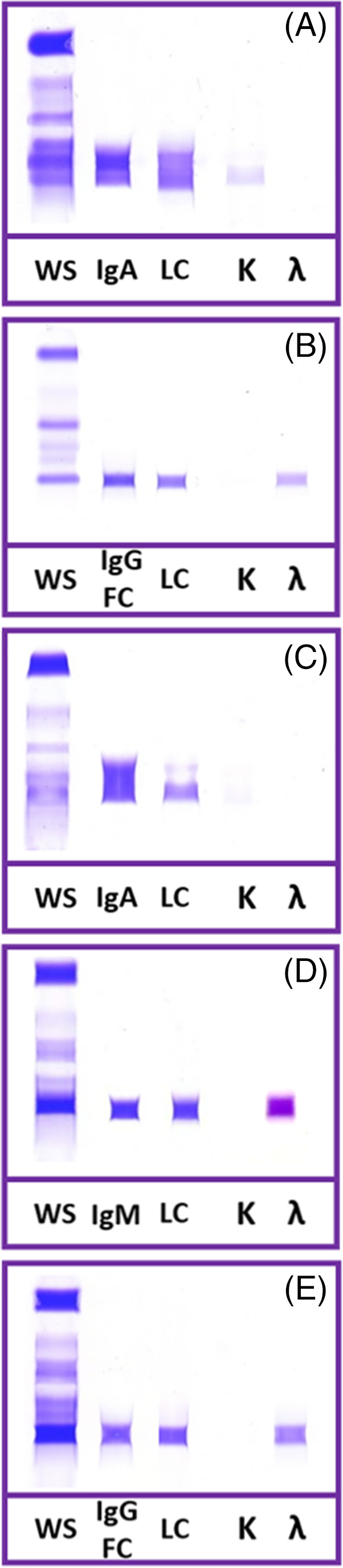
Labeling patterns found when using anti‐human light chain reagents to label canine light chain characterized by liquid chromatography and tandem mass spectrometry. Accurate labeling was observed in several cases, A,B. However, the anti‐κ reagent produced only faint labeling, C, or failed to label, D, some κ‐LC cases and the anti‐λ reagent inaccurately labeled some κ‐LC cases, E. A, Case 10 an IgA : κ case. B, Case 26 an IgG : λ case. C, Case 11 an IgA : κ case. D, Case 8 an IgM : κ. E, Case 3, an IgG : κ

The anti‐κ and anti‐λ antibody labeling on all samples was subjectively graded as negative, faint, or positive. Anti‐human based IF labeling did not match LC‐MS/MS identification (Table 1, Figure 4). Six dogs (3 κ‐LC, 3λ‐LC) failed to label with either anti‐human LC reagents. All remaining λ‐LC dogs had expected labeling with anti‐λ reagent and did not label with anti‐κ reagent. Five κ‐LC cases were labeled only with the anti‐κ reagent. The remaining 2 κ‐LC cases were labeled strongly with anti‐λ; 1 of these did not label with anti‐κ and the other had only faint anti‐κ labeling.

**TABLE 1:** Comparison of labeling pattern of the anti‐human κ (K) and anti‐human λ (L) reagents by immunofixation with canine light chain class as determined by liquid chromatography and tandem mass spectrometry

IF labeling pattern		LC–MS/MS ID	
L	K	L	K
Y	N	9	1
Y	F	0	1
N	Y	0	2
N	F	0	3
N	N	3	3

*Note*: Immunofixation (IF) labeling was graded as positive (Y), faint (F) or negative (N).

Anti‐fλ IF was performed on available serum and urine from all cases with a documented Bence‐Jones proteinuria by electrophoresis. All electrophoresis defined Bence‐Jones protein bands were labeled by the anti‐fλ reagent; none were labeled by the anti‐fκ reagent. An electrophoretically identical restriction that was labeled by anti‐fλ reagent was also present in the paired serum from 5/8 of these cases. The 2 dogs without paired urine had a restriction that was labeled by the anti‐fλ reagent. The 3 dogs with mixed proteinuria, had a γ‐globulin restriction that was labeled by the anti‐fλ reagent but a similarly labeled restriction was not noted in the serum. Close comparison of the fLC and routine IF gels failed to identify a nonheavy chain labeling restricted protein band in the routine LC reagent lane of any of the cases.

**FIGURE 5:**
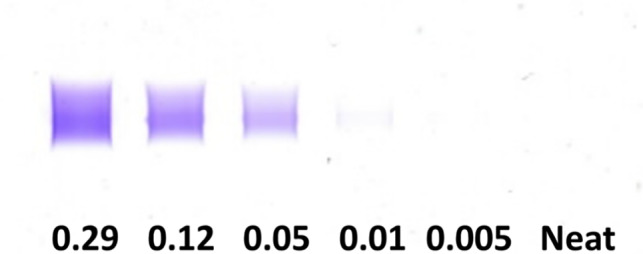
Limit of detection assay using anti‐human free λ light chain. Bence‐Jones proteinuric urine identified as λ‐light chain was spiked into normal canine serum. Immunofixation using an anti‐human free λ light chain produced labeling down to 0.01 g/dL of spiked protein

Using the described protocol, distinct labeling was visible in all tested spiked concentrations down to and including 0.01 mg/dL of fλ‐LC, Figure 5.

## 5 DISCUSSION

Here, we describe a case of LC myeloma in a dog identified by SPE, UPE, and an fLC‐specific IF protocol. The fLC IF protocol is further evaluated in 10 additional cases with sfLC, ufLC, or both, demonstrating detection of fLC in both dogs and cats. Data from 19 dogs with LC‐MS/MS suggest that the tested anti‐human reagents might not distinguish canine λ‐LC from κ‐LC.

Electrophoretic diagnosis of fLC in urine/Bence‐Jones proteinuria has been based on the presence of a restricted protein band in urine which is not present in paired serum.^17^ Five cases had a monoclonal band in the urine which did not correlate with a similar band in the paired serum sample and met the traditional diagnostic criteria of urinary fLC. However, nonimmunoglobulin proteins from tubular and glomerular damage can produce a restricted urinary protein band in electrophoresis of samples that have a Bence‐Jones proteinuria.^11^ In addition, prostatic arginine esterase has a similar molecular weight, ~25 kDa, to fLC and can be found as a restricted protein band in the urine of intact male dogs.^18^ Positive identification of a restricted band as an immunoglobulin subunit is ideal to confirm the diagnosis of urinary fLC.

The electrophoresis‐based diagnosis of Bence‐Jones proteinuria in these cases was confirmed using several methods. First, nrPAGE evaluation confirmed the presence of an appropriately sized protein in the urine. By performing this evaluation under nonreduced conditions, LC present as part of an intact immunoglobulin remained bound to their heavy chains and were retained in larger moieties, allowing distinction of free from bound LC. Degradation of intact LC due to bacterial activity or sample aging can occur but the study samples were stored at −80°C and should not have been degraded. In addition, heavy chain labeling in routine and fLC IF consistently had matching LC labeling using either the LC (routine IF) or fλ (fLC IF) reagents, suggesting that all heavy chain was found in intact immunoglobulins and degradation of intact immunoglobulin was unlikely. The LC bands isolated by PAGE evaluation were identified by LC‐MS/MS as either λ‐LC or κ‐LC with high confidence using species‐specific protein entries. Bence‐Jones proteinuria was confirmed in these cases.

Urine M‐protein concentration in human medicine is ideally expressed as amount of protein excreted in a 24‐hour period.^17^ This requires collection of large amounts of urine. The urinary M‐protein concentration in our samples was calculated as an absolute concentration (ie, g/dL) and as a ratio with creatinine (ie, M‐protein : creatinine ratio). This approach is discussed as a possible method for quantification of M‐protein in a random urine sample and has been used with other urinary protein biomarkers.^17^, ^19^, ^20^ The M‐protein : urine creatinine ratio should compensate the varying degrees of urine concentration and may be useful for quantifying urinary M‐protein in veterinary patients. The clinical utility of this metric in dogs and cats is not known.

The anti‐human fLC IF kit used contains reagents that specifically target obscured antigens that allows identification of human LC. The anti‐κ and anti‐λ reagents are class specific and target antigens accessible when the LC is bound to heavy chain, allowing detection of free and bound LC. The fLC reagents are class specific and target antigens hidden when the LC is bound to heavy chain. Genetic analysis suggests similarity between human and canine LC loci and gene arrangement.^21^ Evaluation of anti‐human heavy chain reagents demonstrated substantial cross‐species labeling.^8^ However, neither of these facts proves that antibodies against human LC would accurately identify all canine or feline LC.^8^, ^21^

To evaluate the performance of anti‐human reagents, canine and feline LC were characterized using LC‐MS/MS and species‐specific databases. While it is possible that the LC‐MS/MS identity of the LC class is incorrect, review of that data suggests a high degree of confidence. Reevaluation of the spectra using several permutations of the database, including the removal of all synthetic peptide entries, produced identical results. The discord between the IF based identity and the LC‐MS/MS‐based identity is likely due to inaccuracies caused by the use of nonspecies‐specific reagents.

There was inconsistent labeling of canine samples by the anti‐κ and anti‐λ reagents, including lack of labeling by both reagents or nonclass‐specific labeling of canine κ‐LC by the anti‐λ reagent. In addition, we speculate that the faint staining of some cases by the anti‐κ reagent is evidence of lower avidity of this reagent to canine κ‐LC. Previous evaluation of anti‐human reagents in an immunohistochemical platform to label canine plasma cells found several cases which did not label with either anti‐κ or anti‐λ reagents, similar to what was found in this work but double labeling was not documented.^22^ The potential effects of low avidity and nonclass‐specific labeling is significant since previous immunohistochemical evaluation of canine LC usage was based on labeling by anti‐human LC reagents only without documented validation of the immunohistochemical methods.^22^, ^23^ Low avidity and inaccurate labeling would call into question the accuracy of the reported approximately 1 : 9 ratio of κ‐LC to λ‐LC in health and disease. Further evaluation of the currently used anti‐human LC immunohistochemistry reagents in the dog is recommended. Ideally, a specific reagent that recognizes canine κ‐LC and λ‐LC would be available.

The anti‐fλ reagent identified all Bence‐Jones proteins and appeared independent of the heavy chain bound LC IF labeling in the serum and urine. This suggests that this anti‐fλ reagent can positively identify only the free form of canine and feline LC. Additionally, the western blot data suggest accurate labeling by anti‐fλ; the known λ‐LC predominant cases had strong labeling with anti‐fλ and the known κ‐LC predominant had strong labeling with anti‐fκ. The κ‐LC predominant sample had lower amounts of λ‐LC documented by LC‐MS/MS, as determined by spectral counts. These lower concentrations of the opposite class would cause the weaker labeling of the anti‐fλ to the κ‐LC predominant sample and would mask any cross‐reactivity of these reagents. There was nonclass‐specific IF labeling by the anti‐fλ reagent of fκ, suggesting that there is some degree of cross‐reactivity. Ideally purified canine λ‐fLC and not native samples would be used to evaluate for cross reactivity, but it is likely that the anti‐fλ antibody accurately labels canine fLC and does not distinguish class. We have not yet found a sample with a diagnosis of Bence‐Jones proteinuria based on paired serum and urine that fails to label with the anti‐human fλ reagent, but it is reasonable to presume that such cases could arise. Until a set of species‐ and class‐ specific anti‐fLC reagents become available, use of the anti‐human fλ reagent to identify but not rule out fLC in the dog and cat seems reasonable.

It is more challenging to explain the performance of the anti‐fκ reagent. This reagent strongly labeled the κ‐LC predominant sample and demonstrated weaker labeling of the λ‐LC predominant samples by western blot, suggesting that it was capable of binding to canine κ‐LC. However, the anti‐fκ reagent did not label any of the fLC samples by IF, including the 2 canine cases with documented Bence‐Jones proteinuria and LC‐MS/MS identified κ‐LC. This might suggest low avidity of this reagent with canine κ‐LC which interferes with the ability to effectively “fix” canine κ‐LC during the IF protocol. Whatever the reason, the anti‐fκ reagent was not useful in confirming the presence of fLC in canine samples. Evaluation of this reagent in additional samples and development of a canine‐specific anti‐fκ reagent is likely needed.

This work demonstrates fLC in the serum of either dogs or cats. Although the SPE profiles of many in our cohort did not contain visually apparent fLC restrictions, the location of the fLC by IF was consistent between the serum and paired urine. The rapid clearance of fLC from serum can explain the lack of electrophoretic findings in the serum and makes diagnosis of serum fLC without the use of fLC IF challenging.^11^ The anti‐fλ reagent used in the described protocol allowed identification of serum fLC by IF with a lower limit of detection of 0.01 g/dL in serum. The reported lower limit of detection of monoclonal immunoglobulins by IF methods in humans is lower, 0.001 g/dL.^17^ The difference in analytical sensitivity of the anti‐human reagent for human and veterinary samples might be due to a lower cross‐species avidity, an effect of the methods used in this study or some other cause.

Several studies have evaluated the clinical significance of Bence‐Jones proteinuria. These studies have been based on the original heat precipitation method for identification of Bence‐Jones proteins, first described over 150 years ago.^24^ This technique is poorly specific with a false positive rate of 20% due to connective tissue disorders, renal disease and nonplasmacytic malignancies.^25^ Nonetheless, available data suggest that Bence‐Jones proteinuria defined by the heat precipitation test is a negative prognostic factor associated with a shorter survival time in the dog.^26^ Similar findings are seen in humans with increased concentrations of fLC secondary to multiple myeloma, which is suspected to be due to the sequalae from overproduction of fLC leading to renal failure.^7^, ^27^, ^28^ Renal failure is more common in LC only myeloma than in other variants of multiple myeloma, but still occurs in multiple myeloma patients who excrete similar quantities of LC.^28^ fLC are described as being nephrotoxic and human medicine suggests the need for rapid reduction of serum fLC concentrations to preserve renal function.^7^, ^29^, ^30^ In the index case, the dog had evidence of renal disease at presentation, which progressed to renal failure. This case presented mildly azotemic and with a normal SDMA but, did not have the highest urinary fLC concentration compared to the other cases, but did develop end‐stage renal failure within months of diagnosis. The clinical importance of fLC production relative to prognosis and outcome in veterinary cases needs to be investigated with current methodology and across lymphoma/myeloma subtypes.

The data here suggest that evaluation of SPE alone is insufficient to diagnose all immunoglobulin secreting tumors in the dog and cat. LC only producing tumors were present in both species. The frequency of occurrence of LC only myeloma in veterinary medicine is unknown but is estimated to be found in 15% to 20% of human multiple myeloma cases.^31^, ^32^ As was seen in many of the cases in this study, approximately 80% of human cases with fLC also have a complete serum M‐protein which produces an electrophoretically abnormal serum profile and allows the diagnosis of a secretory myeloma related disease or at least suggest the need for further evaluation.^33^ Evaluation of sfLC or ufLC IF might not be needed in these cases to make the diagnosis of an immunoglobulin secreting neoplasm. However, evaluation of SPE alone in the remaining 20% of cases fails to detect the M‐protein. Evaluation of paired urine samples could be used to screen for an fLC M‐protein but as cases 6 and 29 demonstrate, not all M‐protein containing urine samples will have a distinct visible restriction by UPE alone.^11^ At Colorado State University's Clinical Pathology Laboratory, we often do not get paired serum and urine samples from cases submitted for M‐protein screening. Based on the current data, it is highly likely that fLC M‐proteins are missed when only SPE is performed, and that LC myeloma is underdiagnosed in veterinary medicine. Serum fLC and ufLC IF could be useful when fLCs are suspected (ie, relatively normal SPE but the patient has proteinuria, positive Bence‐Jones protein urine test heat precipitation test or the suspicion of an immunoglobulin secreting neoplasm). Serum fLC IF could also be useful when concurrent urine is not submitted for electrophoretic evaluation but might not replace electrophoretic and IF evaluation of urine.

This study demonstrates that the human‐targeted anti‐fλ reagent accurately labels fLC in the dog and cat. Based on these results, it appears that these reagents might not accurately distinguish LC class, but it is uncertain if this has any clinical significance. Additional studies evaluating this protocol in a larger cohort to determine the diagnostic sensitivity and specificity of this assay for detecting sfLC and ufLC are needed. Nevertheless, we showed that fLC can be detected in the serum of both dogs and cats and that this test could be a useful diagnostic tool for myeloma‐related disorders.

## CONFLICT OF INTEREST DECLARATION

Authors declare no conflict of interest.

## OFF‐LABEL ANTIMICROBIAL DECLARATION

Authors declare no off‐label use of antimicrobials.

## INSTITUTIONAL ANIMAL CARE AND USE COMMITTEE (IACUC) OR OTHER APPROVAL DECLARATION

Authors declare no IACUC or other approval was needed.

## HUMAN ETHICS APPROVAL DECLARATION

Authors declare human ethics approval was not needed for this study.

## Supporting information


**FIGURE S1** Western blot analysis of the fλ and fκ labeling antibodies used in the human immunofixation kit. Whole serum and urine samples were reduced. Cases 1, 2, and 9 were identified as lambda light chain and case 12 was identified as kappa light chain by mass spectrometry. Faint to strong labeling is noted in all lanes.

**TABLE S1** Cases with history
**TABLE S2**: Labeling antibodies used for canine routine IF, feline routine IF and fLC IF
