# Supplementary figures and images for: Light chain myeloma and detection of free light chains in serum and urine of dogs and cats
Source: J Vet Intern Med. 2021 Feb 26;35(2):1031–40. doi: 10.1111/jvim.16070 (PMC7995444; doi:10.1111/jvim.16070)

## f $\lambda$ antibody:

kDa      L      2S      2U      9S      9U      1S      1U      12S

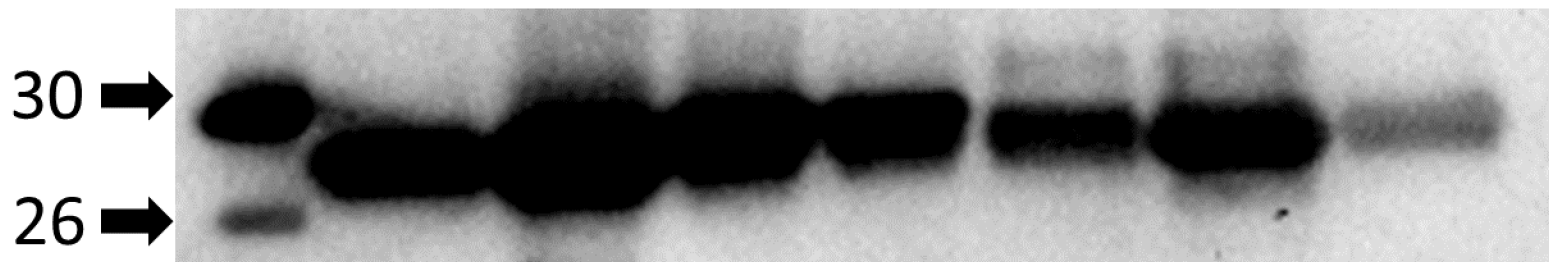

## f $\kappa$ antibody:

kDa      L      2S      2U      9S      9U      1S      1U      12S

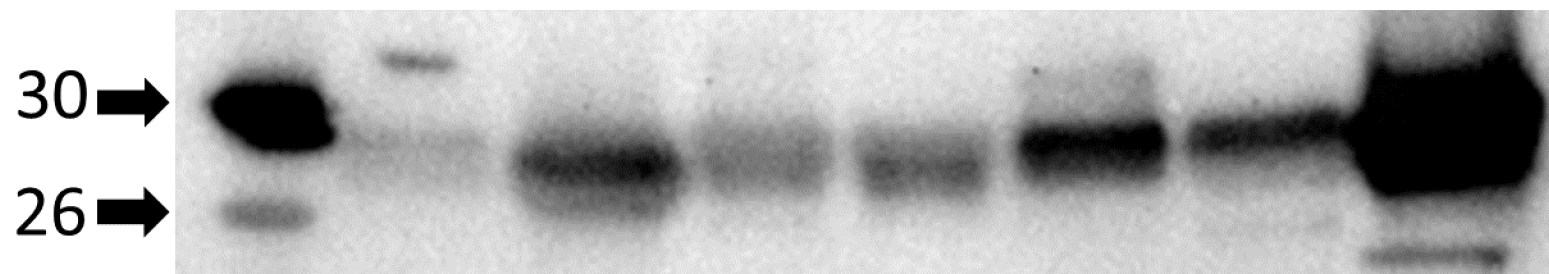

Supplement: Supplementary file 1 — FIGURE S1 Western blot analysis of the fλ and fκ labeling antibodies used in the human immunofixation kit. Whole serum and urine samples were reduced. Cases 1, 2, and 9 were identified as lambda light chain and case 12 was identified as kappa light chain by mass spectrometry. Faint to strong labeling is noted in all lanes. [file JVIM-35-1031-s002.pdf]
