# Supplementary material for: Light chain myeloma and detection of free light chains in serum and urine of dogs and cats
Source: J Vet Intern Med. 2021 Feb 26;35(2):1031–40. doi: 10.1111/jvim.16070 (PMC7995444; doi:10.1111/jvim.16070)
Supplement: Supplementary file 2 — TABLE S1 Cases with history TABLE S2: Labeling antibodies used for canine routine IF, feline routine IF and fLC IF [file JVIM-35-1031-s001.pdf]

Supplemental Table 1: Cases with history (Abbreviations: AST, American Staffordshire Terrier; BG, Biclonal gammopathy; ESS, English Springer Spaniel; F/S, female/spayed; GSP, German Shorthaired Pointer; HG, hypogammaglobulinemia; MAS, Miniature Australian Shepherd; M/I, male/intact; M/N, male/neutered, MG, Monoclonal gammopathy; SPE, serum protein electrophoresis; UP:UC, urine protein:urine creatinine; UPE, Urine protein electrophoresis; WNL, within normal limits)

ID#	Age (years)	Breed	Sex	Species	Total Protein (g/dL):	Urine Total Protein (mg/dL):	UP:UC	SPE	UPE	Involved heavy chain (By IF)
1	8	Mixed	F/S	Canine	6	61.6	0.88	WNL	MG	-
2	8.5	ESS	F/S	Canine	7.6	2130	2.27	BG	MG	IgG/IgM
3	13.5	Mixed	F/S	Canine	9.3	-	-	MG	MG	IgG
4	1	English Bulldog	M/I	Canine	5.9	36	3.68	HG	MG	-
5	12	MAS	M/N	Canine	11.5	1870	1.92	BG	MG	IgA
6	8	Border Collie	M/I	Canine	10.7	127	-	MG	MG	IgM
7	12	Mixed	F/S	Canine	13.9	-	-	MG	N/A	IgG
8	9	Doberman Pinscher	F/S	Canine	10.8	-	-	MG	N/A	IgM
9	14	Mixed	F/S	Canine	9.5	-	-	BG	N/A	IgA
10	10	Labrador Retriever	F/S	Canine	8.6	-	-	BG	N/A	IgA
11	7	Labrador Retriever	F/S	Canine	9.5	-	-	BG	N/A	IgA

12	8	Labrador Retriever	F/S	Canine	9.2	-	-	BG	N/A	IgA
13	8	Mixed	M/N	Canine	8	-	-	BG	N/A	IgA
14	12	Golden retriever	F/S	Canine	7.4	-	-	BG	N/A	IgA
15				Canine	9.9	-	-	MG	N/A	IgM
16	11	AST	M/N	Canine	9.8	-	-	MG	N/A	IgM
17	6	Mixed	M/N	Canine	6.5	-	-	MG	N/A	IgM
18	4	GSP	F/S	Canine	10.1	-	-	MG	N/A	IgM
19	9	Labrador Retriever	M/N	Canine	11.5	-	-	MG	N/A	IgG
20	14	Labrador Retriever	F/S	Canine	8.2	-	-	MG	N/A	IgG
21	8	Cocker Spaniel	M/N	Canine		-	-	MG	N/A	IgG
22	13	Labradoodle	M/N	Canine	10	-	-	MG	N/A	IgG
23	10	Mixed	F/S	Canine	14.6	-	-	MG	N/A	IgG
24	11	Mixed	M/N	Canine	12.9	-	-	MG	N/A	IgG
25	10	Golden Retriever	M/N	Canine	9.2	-	-	MG	N/A	IgG
26	13	Labradoodle	M/N	Canine	10.4	-	-	MG	N/A	IgG
27	9	Airedale	M/N	Canine	11.6	-	-	MG	N/A	IgG

28	14	DSH	M/N	Feline	14.2	228	2.88	MG	MG	IgG
29	5.5	DSH	M/N	Feline	7.3	65	-	HG	Mixed	-

Supplemental Table 2: Labeling antibodies used for canine routine IF, feline routine IF and FLC IF

	Name	Host	Target	Dilution Factor (sera:diluent)	Manufacturer #
Routine Canine	WS	goat	canine whole serum	1:16	A40-108[a]
	IgG-Fc*	goat	canine IgG Fc	1:16	SAB3700101[b]
	IgA	goat	canine IgA	1:8	A40-104A[a]
	IgM	goat	canine IgM	1:8	A40-116A[a]
	LC	goat	canine light chain	1:8	A40-124A[a]
	IgG4	sheep	canine IgG1	1:8	A40-120A[a]
Routine Feline	WS	goat	feline whole serum	1:16	A20-106[a]
	IgG	goat	feline IgG H+L	1:16	A20-115[a]
	IgA	goat	feline IgA	1:8	A20-101A[a]
	IgM	goat	feline IgM	1:8	A20-100A[a]
	LC	goat	feline light chain	1:8	A20-121P[a]
Free Light Chain	Fix	-	protein fixative	1:3	4815[c]
	GAM	rabbit	human IgG, IgA and IgM	1:3	
	κ	rabbit	human free and bound κ light chain	1:3	
	λ	rabbit	human free and bound λ light chain	1:3	
	fκ	rabbit	human free κ light chain	1:2	
	fλ	rabbit	human free λ light chain	1:2	

a = Bethyl Laboratories Inc, Montgomery, TX, USA
b = Sigma-Aldrich, St Louis, MO, USAX
c = Sebia Inc, Norcross, GA, USA
* IgG-Fc antisera diluted with PBS at 1:4
